# MicroRNA-146a Provides Feedback Regulation of Lyme Arthritis but Not Carditis during Infection with *Borrelia burgdorferi*

**DOI:** 10.1371/journal.ppat.1004212

**Published:** 2014-06-26

**Authors:** Robert B. Lochhead, Ying Ma, James F. Zachary, David Baltimore, Jimmy L. Zhao, John H. Weis, Ryan M. O'Connell, Janis J. Weis

**Affiliations:** 1 Division of Microbiology and Immunology, Department of Pathology, University of Utah, Salt Lake City, Utah, United States of America; 2 Department of Veterinary Pathobiology, University of Illinois at Urbana-Champaign, Urbana, Illinois, United States of America; 3 Department of Biology, California Institute of Technology, Pasadena, California, United States of America; Medical College of Wisconsin, United States of America

## Abstract

MicroRNAs have been shown to be important regulators of inflammatory and immune responses and are implicated in several immune disorders including systemic lupus erythematosus and rheumatoid arthritis, but their role in Lyme borreliosis remains unknown. We performed a microarray screen for expression of miRNAs in joint tissue from three mouse strains infected with *Borrelia burgdorferi*. This screen identified upregulation of miR-146a, a key negative regulator of NF-κB signaling, in all three strains, suggesting it plays an important role in the *in vivo* response to *B. burgdorferi*. Infection of B6 miR-146a^−/−^ mice with *B. burgdorferi* revealed a critical nonredundant role of miR-146a in modulating Lyme arthritis without compromising host immune response or heart inflammation. The impact of miR-146a was specifically localized to the joint, and did not impact lesion development or inflammation in the heart. Furthermore, B6 miR-146a^−/−^ mice had elevated levels of NF-κB-regulated products in joint tissue and serum late in infection. Flow cytometry analysis of various lineages isolated from infected joint tissue of mice showed that myeloid cell infiltration was significantly greater in B6 miR-146a^−/−^ mice, compared to B6, during *B. burgdorferi* infection. Using bone marrow-derived macrophages, we found that TRAF6, a known target of miR-146a involved in NF-κB activation, was dysregulated in resting and *B. burgdorferi*-stimulated B6 miR-146a^−/−^ macrophages, and corresponded to elevated IL-1β, IL-6 and CXCL1 production. This dysregulated protein production was also observed in macrophages treated with IL-10 prior to *B. burgdorferi* stimulation. Peritoneal macrophages from B6 miR-146a^−/−^ mice also showed enhanced phagocytosis of *B. burgdorferi*. Together, these data show that miR-146a-mediated regulation of TRAF6 and NF-κB, and downstream targets such as IL-1β, IL-6 and CXCL1, are critical for modulation of Lyme arthritis during chronic infection with *B. burgdorferi*.

## Author Summary

Lyme Disease is caused by infection with the bacteria *Borrelia burgdorferi*, is transmitted through infected deer ticks (*Ixodes scapularis*), and often leads to arthritis that can persist, even after antibiotic treatment. Here, we have identified a microRNA that is critical in modulating Lyme arthritis, but not carditis. This microRNA, miR-146a, is a negative regulator of NF-κB signaling, known to be important in host defense against pathogens, and long suspected to play a role in Lyme arthritis development. Mice lacking miR-146a develop more severe arthritis and show signs of hyperactive NF-κB activation during the persistent phase of infection. Heart manifestations of disease were not altered. Furthermore, this severe arthritis is independent of host defense, since these mice are better able to clear invading bacteria in joints, and bacterial numbers are similar in heart and ear tissue. We identified TRAF6 as an important target of miR-146a-mediated NF-κB regulation of pro-inflammatory cytokines IL-6 and IL-1β, as well as chemokines CXCL1 and CXCL2. Our data demonstrate the importance of maintaining appropriate regulation of amplitude and resolution of NF-κB activation during *Borrelia burgdorferi* infection, and provide a novel model for elucidating the role of NF-κB in Lyme arthritis development, independent of effect on host defense.

## Introduction

Lyme Disease is caused by infection with *Borrelia burgdorferi*, a tick-borne spirochete [1], and is the most common vector-borne disease in the United States with an estimated 300,000 cases per year [2]. Often, infection leads to acute arthritis in humans. Clinical manifestations of Lyme arthritis include inflammatory cell infiltration, edema, synovial hyperplasia and remodeling of bone and connective tissue [3], [4]. In some cases, infection can induce autoimmunity, despite treatment with antibiotics [5]. The reason why arthritis fails to resolve remains poorly understood, but is believed to be the result of dysregulation of host immune response to infection [6].

Several inbred mouse strains exhibit varying degrees of disease severity similar to human patients [7], [8]. Whereas the C57BL/6 (B6) mouse strain develops mild arthritis, C3H and various knockout strains such as B6 IL10^−/−^ mice develop moderate to severe arthritis [7], [9]. Furthermore, the intensity of the inflammatory response for a given spirochete burden varies greatly among strains, implicating host immune response as driving arthritis development [9], [10]. Our laboratory and others have used the mouse model system to elucidate key regulators of host immune response to infection.

Since its discovery, nuclear factor-kappa B (NF-κB) has been identified as a key regulator in many cellular functions including inflammation and cancer [11]. *B. burgdorferi* lipoproteins are extremely potent activators of Toll-like receptor 2 (TLR2)-mediated NF-κB activation and cytokine production, and are important for host defense [12]–[16]. Mice lacking TLR2 or the adapter protein myeloid differentiation primary response gene (88) (MyD88) exhibit a failure to control infection [14], [17]–[21]. Although these knockout studies clearly demonstrate an important role of NF-κB in host defense, elucidating its role in inflammation and Lyme arthritis has remained elusive.

While NF-κB activation is critical in response to infection, downregulation is equally important to avoid excess inflammation, tissue damage and autoimmunity [22]. MicroRNAs (miRNAs) have recently been identified as being important regulators of NF-κB [23] and autoimmunity [24]. These small regulatory RNAs are posttranscriptional regulators of gene expression [25], and one miRNA, miR-146a, has been shown to be a modulator of innate immune response to TLR ligands [26]. Targets of miR-146a include TNF receptor associated factor 6 (TRAF6) and IL-1 receptor associated kinase 1 (IRAK1), adaptor molecules downstream of the MyD88-dependent TLR and cytokine signaling pathways [27]. Importantly, miR-146a itself is upregulated by IL-1β and TLRs, including TLR2, and thus acts as a negative feedback regulator of NF-κB signaling which is required for immune homeostasis *in vivo* [27]–[31].

Aberrant microRNA expression, particularly miR-146a, has been associated with a variety of inflammatory disorders [32]. In systemic lupus erythematosus, a functional variant in the miR-146a promoter is associated with disease risk [33], and abnormally low miR-146a expression has been associated with more severe symptoms [34]. In contrast, rheumatoid arthritis synovial fibroblasts express abnormally high levels of miR-146a [35], [36], while osteoarthritis chondrocytes express variable levels miR-146a, correlating with disease severity [37], [38].

Despite correlative evidence linking aberrant miRNA expression to diseases such as lupus, RA and OA, determining whether miRNAs play an active role in pathogenesis has yet to be elucidated, and to our knowledge, no studies have examined the role of miRNAs in Lyme arthritis. For these reasons, we sought to determine whether changes in miRNA expression contributed to host defense and Lyme arthritis development during *B. burgdorferi* infection.

## Results

### miR-146a is highly upregulated in B6, C3H and B6 IL10^−/−^ mice during infection

**Figure 1:**
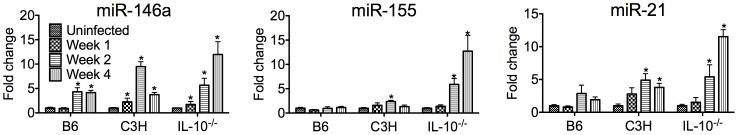
PCR validation of miRNA microarray results. qRT-PCR analysis of miR-146a, miR-21 and miR-155 expression normalized to 5S rRNA from *B. burgdorferi*-infected joints of B6, C3H and IL10^−/−^ mice at 1, 2, or 4 weeks post-infection (n = 3–4). Shown is fold change in expression compared to uninfected controls ± SEM. * indicates statistically significant increase in expression vs. uninfected controls by ANOVA followed by Dunnett's post-hoc test, α = 0.05.

MicroRNA dysregulation has been associated with a number of inflammatory disorders, and we hypothesized that these may play an important role in response to *B. burgdorferi* infection and Lyme arthritis development. We therefore performed a genome-wide screen of changes in miRNA expression in joints of B6, C3H and B6 IL-10^−/−^ mice infected with *B. burgdorferi* at one and two weeks post-infection using an Agilent mouse microRNA microarray (Table 1, Table S1). MicroRNAs differentially regulated included many that have been identified previously as important regulators of immune function. Interestingly, each infection model had a unique miRNA expression “signature,” and we found that only a few dozen miRNAs showed changes in expression during infection. Most of these changes were in C3H mice, and may be due to both differences in inflammatory response and intrinsic differences in miRNA function between strains. At two weeks post-infection, two miRNAs, miR-21 and miR-146a, both induced by NF-κB and associated with TLR signaling, were the most highly upregulated in all three strains (Table 1), and were confirmed using qRT-PCR (Figure 1). Furthermore, these miRNAs maintained high expression, even at 4 weeks post-infection. Interestingly, miR-155 was significantly upregulated in B6 IL10^−/−^ mice, but not in B6 or C3H mice. This microRNA is a proinflammatory NF-κB-induced miRNA associated with T cell-dependent inflammation and autoimmunity [39]–[41], and expression is suppressed by IL-10 [42].

**Table 1:** MicroRNAs most highly changed in expression, based on microarray, in joints of different mouse strains.

B6		C3H		IL-10^−/−^	
miRNA	Fold Δ	miRNA	Fold Δ	miRNA	Fold Δ
miR-146a	5.67	miR-146a	10.8	miR-21	6.08
miR-21	3.92	miR-21	8.12	miR-146a	5.34
miR-706	3.47	miR-142-3p	6.54	miR-155	5.12
miR-29b	2.31	miR-142-5p	4.99	miR-193b	−3.04
miR-715	2.24	miR-34a	4.4	miR-150	−2.56
miR-340-5p	2.21	miR-18a	3.35	miR-145	−2.43
miR-34a	2.16	miR-19b	3.08	miR-181a	−2.37
miR-689	2.12	miR-145	−3.07	miR-181b	−2.29

List of 8 most highly differentially expressed miRNAs in *B. burgdorferi*-infected joints of B6, C3H and B6 IL-10^−/−^ mice at two weeks post-infection, based on Agilent mouse miRNA microarray. Shown is fold-change in expression compared to uninfected controls. Significance was determined using Welch's t-test with Benjamini and Hochberg correction (p<0.05, n = 3–4 mice per group).

Of these, miR-146a was of particular interest, given recent reports showing a link between miR-146a and susceptibility to a variety of inflammatory disorders. Targets of miR-146a, IRAK1 and TRAF6 [27], are involved in TLR2/NF-κB activation, which is an important pathway in controlling *B. burgdorferi* infection [13], [14], [17]–[21]. Also, the observation that miR-146a was upregulated in all three strains suggested that this miRNA likely plays a general role in regulating the immune response to *B. burgdorferi*. For these reasons, our focus turned to studying miR-146a. A B6 miR-146a^−/−^ knockout mouse was recently generated [28], which provided a powerful tool to evaluate the role of miR-146a in mildly arthritic B6 mice. While miR-146a was also upregulated in arthritis-susceptible C3H mice, we suspected that other genetic factors play a dominant role in arthritis development, including excessive Type I IFN production [43], [44] and accumulation of undigested glycosaminoglycans in joint tissue [45]. These effects may limit the ability of miR-146a to modulate arthritis development in the C3H mouse model. It is tempting to speculate, however, that lack of miR-146a in the arthritis-susceptible C3H mouse would lead to even more severe arthritis, as has been reported in the C3H IL-10^−/−^ mouse model [46].

### Impact of miR-146a on Lyme arthritis, carditis and host defense

**Figure 2:**
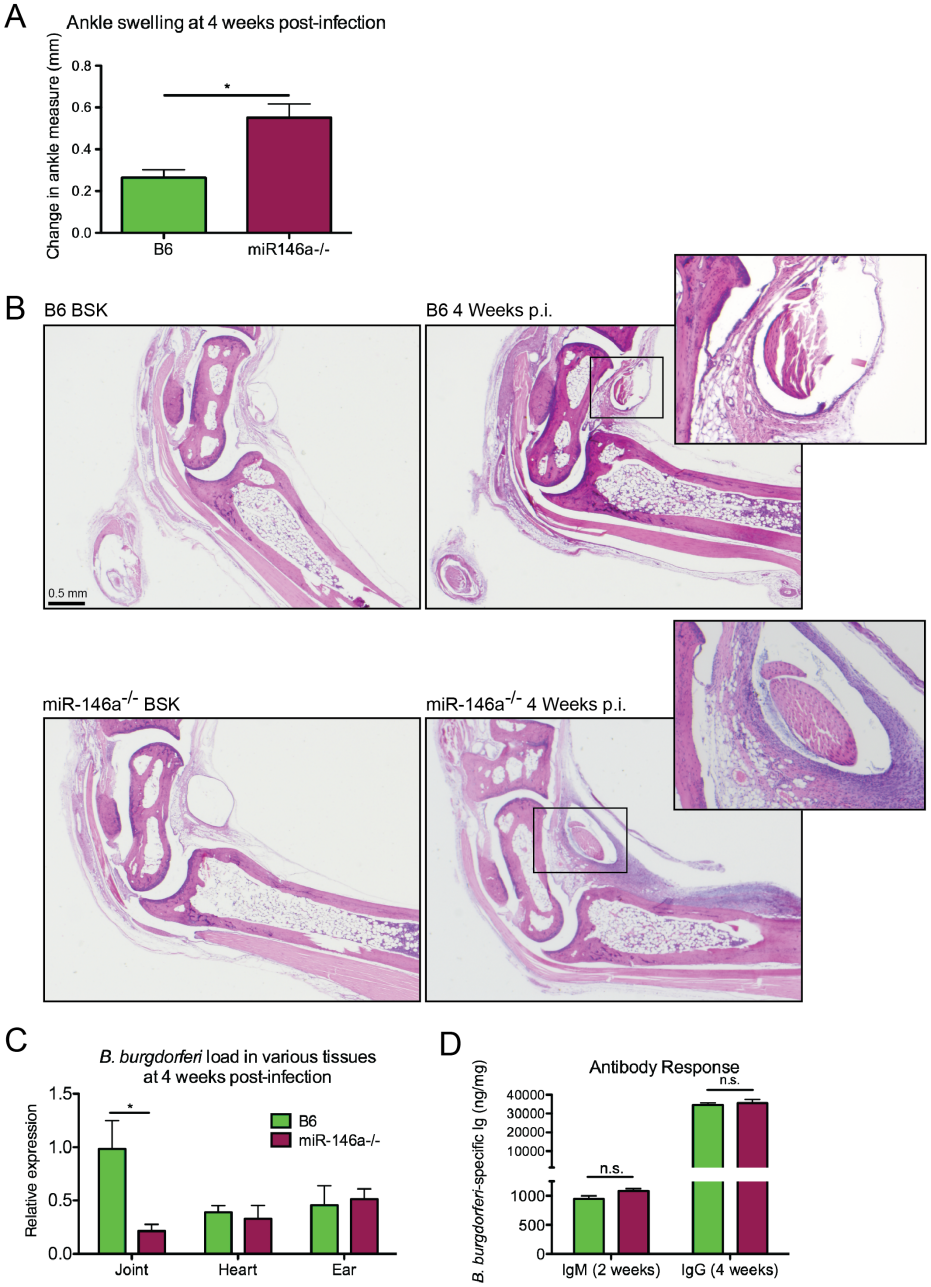
B6 miR-146a^−/−^ mice develop more severe arthritis at 4 weeks post-infection independent of bacterial burden. Arthritis severity was determined for *B. burgdorferi*-infected B6 or B6 miR-146a^−/−^ mice at 4 weeks post-infection. (A) Blinded measurements of rear ankles of mice were taken before infection and at 4 weeks post-infection, and change in ankle measurement is shown. (B) Representative images of H&E-stained tibiotarsal joints from BSK-injected (control) and 4 week-infected B6 and B6 miR-146a^−/−^ mice used for histopathology scoring (see Table 2). Cranial tibial tendons of infected joints are enlarged to show detail of tendon sheath thickening and PMN infiltrate. (C) Bacterial burden was determined by quantifying *B. burgdorferi*-specific *16S rRNA* normalized to 1000 *β-actin* for joint and heart tissue, and *recA*, normalized to 1000 *nidogen* for ear tissue. Pooled from two independent infection experiments (n≥9 mice per experiment) for joints, and from one experiment for heart and ear tissue (n = 5 mice). Statistical significance was determined by Student *t* test (*p<0.01). (D) Antibody concentrations were estimated in serum collected from *B. burgdorferi*-infected B6 and B6 miR-146a^−/−^ mice as described in materials and methods. IgM was measured at 2 weeks post-infection (n = 4) and IgG was measured at 4 weeks post-infection (n = 9–10). Data are representative of 2 independent experiments (n.s.: no significant difference between strains).

Since miR-146a is an important negative regulator of NF-κB activation, we hypothesized that a B6 mouse deficient in miR-146a would develop more severe arthritis during infection with *B. burgdorferi* compared to WT controls. To avoid age-related pathologies associated with B6 miR-146a^−/−^ mice [30], we used 6–8 week-old mice, which is also the age of optimal arthritis in other mouse strains. Arthritis was assessed in *B. burgdorferi*-infected B6 and B6 miR-146a^−/−^ mice. At four weeks post-infection, B6 miR-146a^−/−^ mice developed significantly more severe arthritis. Several markers of arthritis were elevated in B6 miR-146a^−/−^ mice, including ankle swelling (Figure 2A), number and severity of lesions observed, polymorphonuclear (PMN) cell infiltrate, reactive/reparative score (periosteal hyperplasia and new bone formation and remodeling), and tendon sheath thickness (Table 2). Cranial tibial tendon is enlarged in Figure 2B. Control (BSK-injected) mice showed no significant arthritis in either strain, and no significant difference between strains was seen in mononuclear cell infiltrate into inflammatory processes. Importantly, B6 miR-146a^−/−^ mice did not display overwhelming numbers of bacteria; rather, they tended to have somewhat fewer bacteria in infected joints and similar burden in infected heart and ear tissue, as measured by *B. burgdorferi*-specific*16S rRNA* normalized to *β-actin* in joints and heart, and *recA* normalized to mouse *nidogen* in ear tissue (Figure 2C). This difference in bacterial load in joint tissue was likely not due to differences in antibody response, since *B. burgdorferi*-specific IgM and IgG levels were similar between the two strains at two weeks and four weeks post-infection, respectively (Figure 2D). While this does not rule out the possibility that different borrelial proteins could be opsonic targets in the two strains, these data support the notion that increased arthritis observed in B6 miR-146a^−/−^ mice was likely due to a defect in regulation of host immune function rather than compromised host defense. In fact, the decrease in *16S rRNA* in B6 miR-146a^−/−^ mouse joints indicated that arthritis development was independent of bacterial density. This increased arthritis severity with accompanying decreases in bacterial burden is also observed in the B6 IL10^−/−^ mouse model of arthritis [9], [47], [48], and is believed to be due primarily to enhanced innate immune responses [49].

**Table 2:** Histopathology scores of arthritis severity for B6 and B6 miR-146a^−/−^ mice.

Strain	Overall Lesion	PMN Infiltrate	Mono-nuclear Infiltrate	Sheath Thickness	Reactive-Reparative	Total Score
B6	1.53 (.21)	1.05 (.25)	0.58 (.14)	1.42 (.22)	0.26 (.15)	4.84 (.79)
miR-146a^−/−^	**2.84 (.26)**	**2.21 (.26)**	0.37 (.11)	**2.74 (.26)**	**1.58 (.25)**	**9.74 (.97)**

Histopathology scores of rear ankle joints of B6 and B6 miR-146a^−/−^ mice infected with *B. burgdorferi* for 4 weeks. Scores of 0–5, with 5 being most severe, were assigned to each sample. Total score is the sum of scores from each category. Values shown are the mean (±SE), and bold numbers indicate statistically significant difference between strains using Mann-Whitney *U* test (p<0.01), pooled from two independent infection experiments (n = 9–10 each).

**Figure 3:**
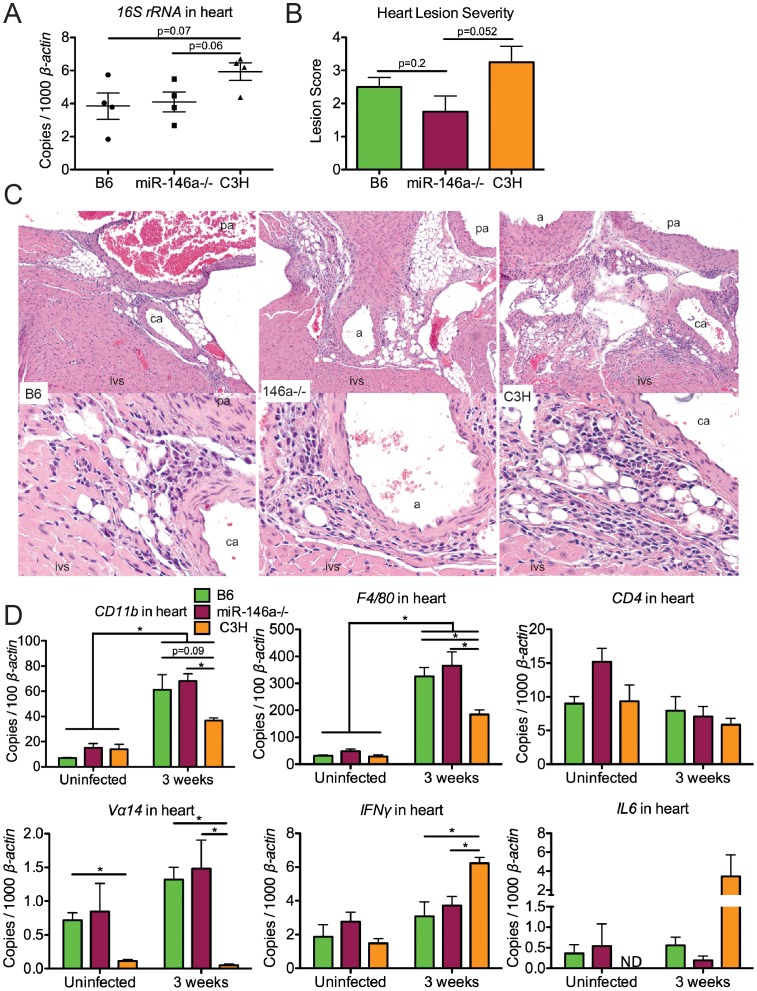
B6 and B6 miR-146a^−/−^ mice have similar *B. burgdorferi* burden and similar levels of inflammation in heart tissue, distinct from C3H mice. Mice were infected with *B. burgdorferi* for 3 weeks, after which hearts were collected for analysis of bacterial burden and inflammation. (A) Bacterial presence was quantified with *B. burgdorferi*-specific *16S rRNA*, normalized to 1000 *β-actin* by qRT-PCR. (B) Carditis was blindly assessed using histopathologic evaluation to score lesion severity in heart tissue from B6, B6 miR-146a^−/−^ and C3H mice infected with *B. burgdorferi* for 3 weeks. (C) H&E-stained sections of infected B6, B6 miR-146a^−/−^, and C3H hearts (10× magnification top row, 40× magnification bottom row), with the intraventricular septum (ivs), coronary artery (ca), pulmonary artery (pa) and aorta (a) labeled as indicated. The inflammatory response in these lesions was a mixture of neutrophils and mononuclear inflammatory cells such as lymphocytes and macrophages. (D) Transcripts for *CD11b*, *F4/80*, *CD4* and *Vα14*, *IFNγ* and *IL6* were measured by qRT-PCR, normalized to *β-actin*. 4 mice were used for each strain, and statistically significant difference between groups by ANOVA followed by Tukey's post-hoc test are indicated (*p<0.05). For lesion scoring, Mann-Whitney *U* test was used to determine whether there were statistical significant differences between groups, with p value indicated (n = 4 mice per strain).

In addition to joints, the heart is another target of *B. burgdorferi* infection in mice. We therefore looked for evidence of miR-146a modulating inflammation in heart tissue. Mice are susceptible to Lyme carditis in an MHC-independent manner, and exhibit variation in disease severity, with C3H mice harboring a greater number of bacteria and developing more severe carditis and B6 mice being resistant and harboring fewer bacteria [7], [50], [51]. Lyme carditis is also observed in humans, and although rare, can be fatal [52]. To assess the role of miR-146a in modulating heart inflammation, B6, B6 miR-146a^−/−^ and C3H mice were infected with *B. burgdorferi* for 3 weeks and hearts were removed and assessed for bacterial numbers and changes in transcripts of inflammatory genes. As was seen at 4 weeks post-infection (Figure 2C), bacterial burden, as measured by qRT-PCR analysis of *B. burgdorferi 16S rRNA*, was similar between B6 and B6 miR-146a^−/−^ mice, and both trended lower than what was seen in heart tissue from C3H mice (Figure 3A). Lesions in the heart were also scored for carditis in B6, B6 miR-146a^−/−^ and C3H mice at 3 weeks post-infection. Overall lesion scores were similar in B6 and B6 miR-146a^−/−^ mice, and both trended lower than lesion severity in C3H mice (Figure 3B).

Lesions in hearts at 3 weeks post-infection were characterized by acute to subacute vasculitis/perivasculitis (see Figure 3C) of the 1) microvasculature (capillaries) at the base of the heart (in heart muscle) where the aorta and pulmonary arteries arise, 2) microvasculature (capillaries) within connective tissues supporting these arteries, and 3) microvasculature (capillaries) of the vasa vasorum of the aorta and pulmonary arteries. These lesions affected the vascular system, rather than being primary lesions of the heart muscle (myocarditis). The character and pattern of distribution of these lesions suggested that inflammation of the microvasculature is the result of some type of localized target cell (i.e., endothelium) or target substance (i.e., bacteria) specificity for this location consistent with *Borrelial* adhesin-host ligand binding within the vascular endothelium [53], [54]. Vascular turbulence or oxygen concentration could also be involved.

Lyme carditis is associated with macrophage infiltration [50], and invariant NKT cells have been shown to play a protective role in B6 mice [55]. We therefore used PCR analysis of macrophage (CD11b and F4/80) and NKT cell (CD4 and Vα14) markers to assess changes in cellularity in infected heart tissue (Figure 3D), as performed previously [56], [57]. Significant upregulation of macrophage markers *CD11b* and *F4/80* was observed in all three strains, as expected based on previous research [50]. The magnitude of upregulation was not different between B6 and miR-146a deficient mice, indicating no role of miR-146a feedback on inflammation in this tissue. Interestingly, while *CD11b* and *F4/80* transcripts were also significantly upregulated in C3H mice, the degree of upregulation was somewhat less than upregulation observed in B6 and B6 miR-146a^−/−^ mice at 3 weeks post-infection. Because these data are from whole heart tissue, they reflect cumulative changes in myeloid cell numbers from the entire heart, including changes in resident cardiac macrophages [58], which may dilute out lesion-specific changes identified by histopathology.

*CD4* transcript levels were not significantly different between strains, but while both B6 and B6 miR-146a^−/−^ mice contained similar levels of *Vα14* that trended higher at 3 weeks, C3H mice had very low levels of this transcript in both uninfected and infected heart tissue. This is consistent with significant variation of NKT cell numbers between different mouse strains [57]. PCR analysis was also used to determine changes in expression of various inflammatory cytokines and chemokines in heart tissue (Figure 3D). As previously reported, C3H mice had elevated levels of *IFN*γ transcripts in infected heart tissue [59], which was significantly higher than *IFN*γ mRNA in B6 and B6 miR-146a^−/−^ hearts. This trend was also observed for *IL6* transcripts, although there was significant variation in expression within C3H mice. No differences among B6, B6 miR-146a^−/−^ and C3H mice were observed for transcripts of *IL1β*, *TNFα*, *Cxcl1*, *Cxcl2*, *Ccl2*, *IL10* or *IL12* (data not shown). Together, these data suggest that the nature of host defense, macrophage and NKT cell proliferation and infiltration, as well as cytokine and chemokine expression, is very similar in infected B6 and B6 miR-146a^−/−^ heart tissue, but is quite distinct from observations in carditis-susceptible C3H mice. Fundamental differences between strains have been reported previously in Lyme carditis studies comparing the effect of Stat1 [60] and Ccr2 [51] deficiencies on carditis-susceptible and carditis-resistant mouse strains. Together, these data show that while strain-specific variables influence differences in carditis susceptibility between B6 and C3H mice, miR-146a has no impact on carditis severity in B6 mice.

### B6 miR-146a^−/−^ mice exhibit hyperactive expression of NF-κB target cytokines and chemokines at 4 weeks post-infection

**Figure 4:**
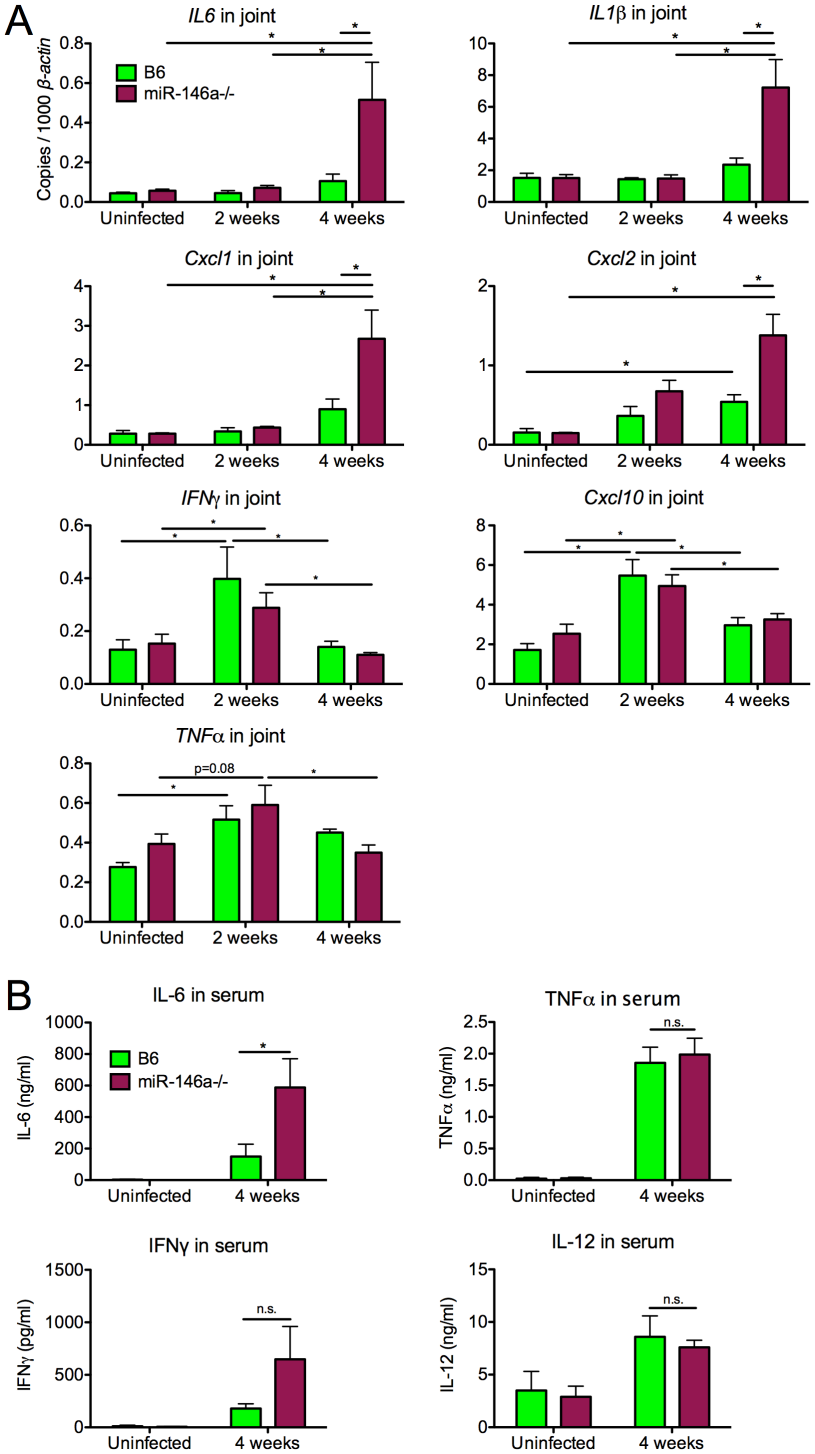
B6 miR-146a^−/−^ mice exhibit hyperactive expression of a subset of NF-κB target cytokines and chemokines at 4 weeks post-infection. (A) RNA was isolated from *B. burgdorferi*-infected rear ankle joints at 2 and 4 weeks post-infection and transcript levels of *IL6, IL1β, Cxcl1, Cxcl2, IFNγ, Cxcl10 and TNFα* were measured by qRT-PCR, normalized to *β-actin*. (B) Serum from *B. burgdorferi*-infected mice was collected by cheek bleed at 4 weeks post-infection, and IL-6, TNFα, IL-12 and IFNγ protein concentration was measured by ELISA. Statistically significant differences between groups by ANOVA followed by Tukey's post-hoc test are indicated (*p<0.05, n≥9 mice per group).

Because miR-146a is known to negatively regulate NF-κB activation, we compared transcripts of genes upregulated by NF-κB in infected joint tissue from B6 and B6 miR-146a^−/−^ mice at 2 and 4 weeks post-infection. We observed that a number of NF-κB inducible genes were significantly elevated at 4 weeks post-infection (but not at 2 weeks) by qRT-PCR in B6 miR-146a^−/−^ joints, compared to WT, including cytokines *IL-1β* and *IL-6*, as well as neutrophil chemokines *Cxcl1* and *Cxcl2* (Figure 4A). CXCL1 has been shown to be required for full arthritis development in C3H mice [61], [62], and increased expression of this gene in B6 miR-146a^−/−^ mice could be directly contributing to arthritis development through recruitment of neutrophils. This is supported by the increase in PMN infiltrate seen at 4 weeks by histopathology, shown in Table 2. Elevated *IL-1β* transcript level is also particularly interesting, since the IL-1 receptor (IL-1R) uses the same adaptors as TLR2/1 for signal transduction, is strongly upregulated by myeloid cells during phagocytosis of *B. burgdorferi* [63], and is dependent on IRAK1 and TRAF6, two miR-146a targets [27]. Furthermore, IL-1β stimulates miR-146a upregulation *in vitro* [27], suggesting that this miRNA negatively regulates IL-1β signaling. It is important to note that uninfected B6 miR-146a^−/−^ mice did not exhibit any abnormalities in expression of these genes, indicating that this hyperactivity is due to a failure to down-regulate the NF-κB response after infection, rather than general NF-κB hyperactivity, as is observed in aging B6 miR-146a^−/−^ mice [30].

Only a distinct subset of inflammatory cytokines and chemokines (*IL-1β, IL-6, Cxcl1, Cxcl2*) appeared to be dysregulated in B6 miR-146a^−/−^ joints. Transcript levels of other Lyme arthritis-associated genes (*IFNγ, Cxcl10, TNFα*) were very similar between the two strains (Figure 4A), and showed a peak in expression at 2 weeks post-infection, followed by resolution at 4 weeks. This is in contrast to arthritis-susceptible B6 IL10^−/−^ mice, where previously published data show that in addition to upregulation in *IL-1β*, *IL-6*, *Cxcl1* and *Cxcl2*, *IFNγ* and *Cxcl10* are upregulated 16-fold and 141-fold at 2 weeks, and 22-fold and 189-fold at 4 weeks, respectively [47]. These data together indicate that the B6 miR-146a^−/−^ mouse is distinct from the B6 IL10^−/−^ model, which is associated with a dramatic IFNγ signature in joints and elevation of IFNγ in serum at 4 weeks p.i. [47], [48].

In order to determine whether there was systemic dysregulation of NF-κB-inducible cytokines, serum was collected from *B. burgdorferi*-infected mice at 4 weeks post-infection and cytokine levels were measured by enzyme-linked immunosorbent assay (Figure 4B). B6 miR-146a^−/−^ mice contained higher levels of IL-6 at 4 weeks post-infection, compared to wild-type, consistent with observations in joint tissue. TNFα and IL-12 serum levels were very similar between strains, and although levels of IFNγ varied widely in B6 miR-146a^−/−^ mice, they were not significantly greater than B6 levels.

### Effect of miR-146a in cell populations isolated from joints early in infection

**Figure 5:**
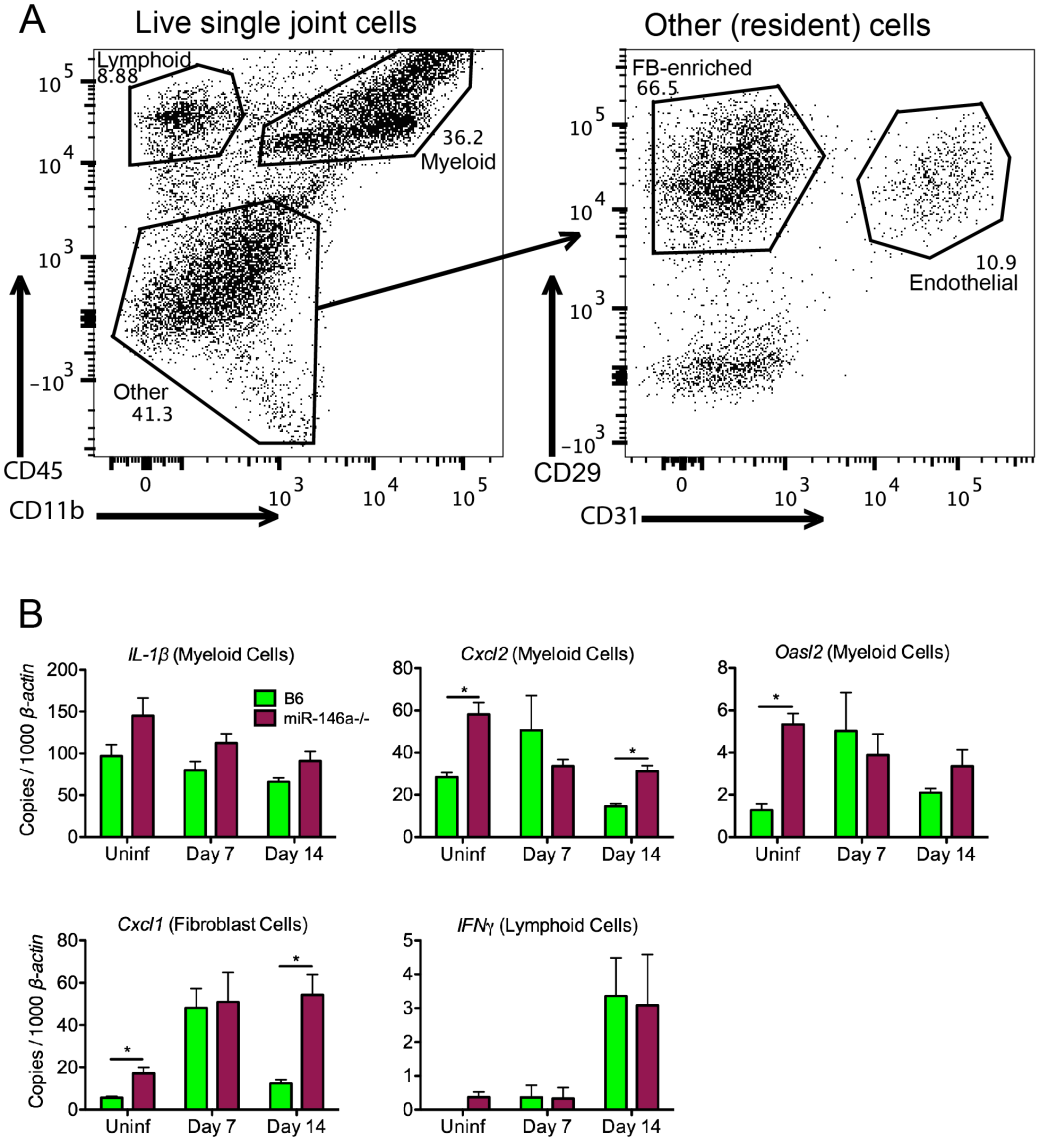
Effect of miR-146a in isolated joint cell populations early in infection. (A) FACS analysis of cells released from joint tissue of B6 mice uninfected or infected with *B. burgdorferi* for 7 or 14 days. Single-cell suspensions of tissue were prepared as described in materials and methods, and cells were stained according to myeloid (CD45+ CD11b+), lymphoid (CD45+ CD11b−), endothelial (CD45− CD31+) or fibroblast-enriched (CD45− CD31− CD29+) lineages, following gating to exclude debris, dead cells and cell doublets. (B) Cells sorted into various lineages depicted in (A) were analyzed for transcript levels of *Cxcl2*, *IL1β* and *Oasl2* in myeloid cells, *Cxcl1* in fibroblast-enriched cells, and *IFNγ* in lymphoid cells, normalized to *β-actin*. Statistically significant differences between groups by ANOVA followed by Tukey's post-hoc test are indicated (*p<0.05). Data are representative of two independent experiments (n = 4 mice for each group).

To identify the effect of miR-146a in various joint cell populations during the early phase of infection *ex vivo*, we digested joints with purified collagenase to release cells into a single-cell solution in order to identify and isolate cell fractions based on lineage markers, including CD45 for leukocytes, CD11b for myeloid cells, CD31 for endothelial cells, and CD29 for fibroblast-enriched cells (Figure 5A). This method has been used in C3H mice to identify cellular sources of genes associated with the arthritogenic Type I IFN response early in infection, and is a sensitive assay to observe cell type-specific effects *ex vivo* that might be missed using whole joint tissue [43]. Using this method, we were able to determine the effect of miR-146a on specific cell types early in infection (Figure 5B). Levels of *IL-1β*, while trending higher in myeloid cells isolated from B6 miR-146a^−/−^ mice, were not significantly different between the two strains. In B6 mice, three genes, *Cxcl2* and the IFN-inducible gene *Oasl2* (in myeloid cells) and *Cxcl1* (in fibroblasts), tended to peak in expression at Day 7 post-infection. In contrast, transcripts were somewhat higher in uninfected B6 miR-146a^−/−^ cell fractions vs. WT, and remained elevated throughout infection. This suggests that B6 miR-146a^−/−^ mice may be poised to initiate a hyperactive immune response. There was no difference in lymphoid *IFNγ* expression between strains, which peaked at Day 14 post-infection, as was seen in whole joint tissue (Figure 4A). It is important to note that, unlike published observations in C3H mice [43], B6 miR-146a^−/−^ mice did not exhibit a robust induction of IFN-responsive genes, such as *Oasl2*, in fibroblasts or endothelial cells at Day 7 post-infection (data not shown). Overall, these data, combined with data from Figure 4, suggest that a number of NF-κB-inducible genes in B6 miR-146a^−/−^ mouse joints are poised for hyper-activation prior to infection, and peak at 4 weeks post-infection, indicating that miR-146a acts to resolve the inflammatory response late in infection, rather than limiting the amplitude of inflammation during early stages of infection.

### Myeloid cell recruitment is increased in infected joints of B6 miR-146a^−/−^ mice

**Figure 6:**
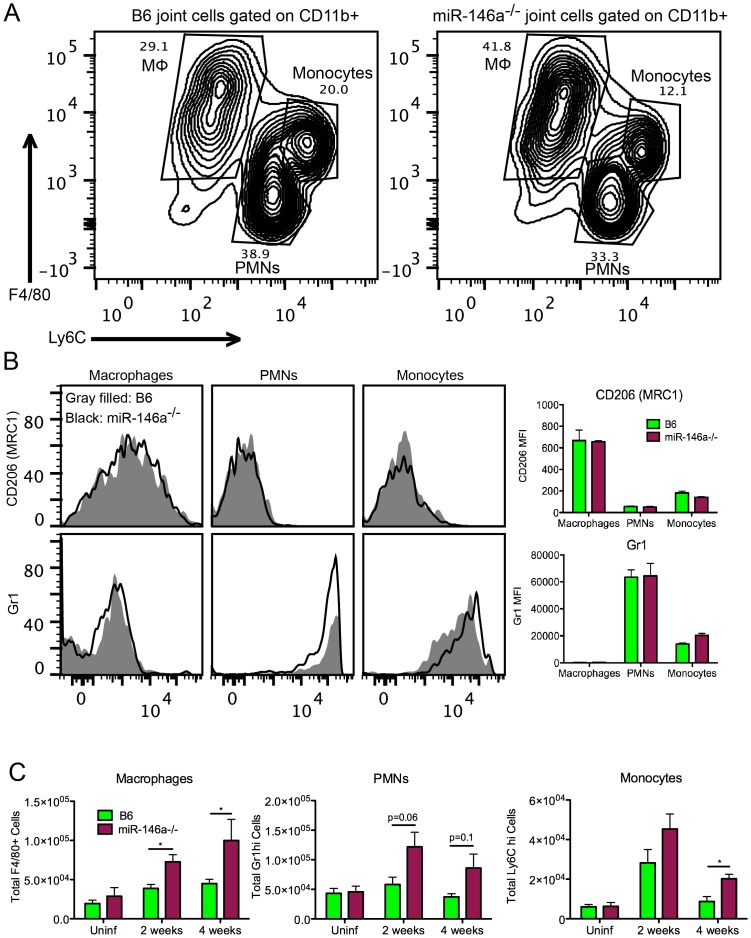
Myeloid cell recruitment is increased in infected joints of B6 miR-146a^−/−^ mice. (A) Representative flow cytometry analysis of CD11b+ cells released from joint tissue of B6 or B6 miR-146a^−/−^ mice infected with *B. burgdorferi* for 2 or 4 weeks, following gating to exclude debris, dead cells and cell doublets. Numbers indicate gate percentages. (B) Representative histogram showing MRC1 (Mannose Receptor, C type 1, CD206) and Gr1 fluorescence intensity in macrophages (Mφ), polymorphonuclear cells (PMNs) and monocyte gates, as shown in (A). Gray shaded area indicates B6 mice and the black line indicates B6 miR-146a^−/−^ mice. Bar graph on right shows average mean fluorescence intensity of macrophages, PMNs and monocytes isolated from 14 day-infected B6 or miR-146a^−/−^ mouse joints (n = 4). (C) Total numbers of myeloid cell populations in joints of uninfected or *B. burgdorferi*-infected mice at 2 and 4 weeks post-infection from B6 or B6 miR-146a^−/−^ mice. Cell populations were identified by flow cytometric analysis with macrophages defined as CD11b+ F4/80+ Ly6C^lo^, polymorphonuclear cells (PMNs) as CD11b+ Gr1^hi^ Ly6C^int^, and monocytes as CD11b+ Gr1^int^ Ly6C^hi^. Statistically significant differences between groups by ANOVA followed by Tukey's post-hoc test are indicated (*p<0.05). For selected values not reaching statistical significance cutoff, p values are listed. Representative of 2 independent experiments (n = 4 for each group).

During cell sorting, we observed differences in cellular infiltrate, particularly in the myeloid cell lineages, in joints during infection. Therefore, a more rigorous analysis of myeloid cells recruited to the joint by flow cytometry was performed at various times during infection. Using joint cell isolation methods described in Figure 5, myeloid cells were characterized from infected joints at 2 and 4 weeks post-infection using fluorescently labeled antibodies against CD11b, F4/80, Ly6C, Gr1 and CD206. CD11b+ myeloid cell populations clustered roughly into three populations, F4/80+ Ly6C^lo^ macrophages, Gr1^hi^ Ly6C^int^ PMNs and Gr1^int^ Ly6C^hi^ monocytes (Figure 6A&B). Furthermore, F4/80+ Ly6C^lo^ macrophages expressed variable levels of CD206 (MRC1, Mannose Receptor C type 1), a marker of alternatively activated (M2-like) macrophages [64]. There was little difference between strains in mean fluorescence intensity (MFI) of MRC1 and Gr1 in each myeloid subpopulation, suggesting that they were phenotypically similar populations. However, while the number of these three myeloid populations in B6 mouse joints changed only modestly in B6 mice, myeloid cell numbers in B6 miR-146a^−/−^ joints were significantly elevated at both 2 and 4 weeks post-infection (Figure 6C). An increased trend in PMN infiltration in B6 miR-146a^−/−^ mice is also consistent with histopathology data shown in Table 2, despite the propensity of PMNs to lyse during enzymatic digestion of joint tissue, resulting in some sample-to-sample variation. Interestingly, there was little difference between strains in infiltrating lymphoid cells at 2 or 4 weeks post-infection (Figure S1). These data, as well as the observation of similar *B. burgdorferi*-specific antibody levels (Figure 2D), suggest that arthritis and host defense phenotypes observed in B6 miR-146a^−/−^ mice shown in Figure 2 and Table 2 are driven primarily by myeloid cells.

### Bone marrow-derived macrophages from B6 miR-146a^−/−^ mice are hyper-responsive to *B. burgdorferi* and have elevated protein levels of TRAF6

The data from Figure 6 implicated myeloid cells as contributors of arthritis development in B6 miR-146a^−/−^ mice. We therefore turned to bone marrow-derived macrophages (BMDMs) to elucidate the molecular mechanism of miR-146a regulation of NF-κB during *B. burgdorferi* infection. BMDMs were cultured from bone marrow extracted from B6 or B6 miR-146a^−/−^ mice and treated with *B. burgdorferi* for 6 and 24 hours. We then measured transcripts of *IL1β*, *IL6* and *TNFα* (Table 3). Transcripts of *IL1β* were approximately 4-fold higher in B6 miR-146a^−/−^ BMDMs, vs. WT, at both 6 and 24 hours, and *IL6* levels were 7.5-fold higher at 6 hours and 2.5-fold higher at 24 hours post-stimulation. Interestingly, *TNFα* transcripts were only 20–30% higher in B6 miR-146a^−/−^ BMDMs, compared to WT. Transcripts for all three cytokines were very low in uninfected cells, and were similar between the two strains (data not shown). This suggests that miR-146a effect on *IL1β* and *IL6* regulation is greater than its effect on *TNFα* expression.

**Table 3:** mRNA expression of induced cytokines in BMDMs from B6 and B6 miR-146a^−/−^ mice after 6 and 24 hours stimulation with *B. burgdorferi*.

Strain	6 hours			24 hours		
	*IL1β*	*IL6*	*TNFα*	*IL1β*	*IL6*	*TNFα*
B6	56.2±2.5	0.008±0.002	127.3±6.6	109.7±12.7	1.07±0.46	6.42±0.58
miR-146a^−/−^	236±8.7	0.06±0.007	168.2±14.7	446.8±14.0	2.67±0.35	7.91±0.37
**Fold Δ**	**4.2**	**7.5**	**1.3**	**4.1**	**2.5**	**1.2**

Induced transcript levels of *IL1β*, *IL6* and *TNFα* (normalized to 1000 *β-actin*) in B6 and B6 miR-146a^−/−^ BMDMs stimulated with *B. burgdorferi* for 6 or 24 hours. Data shown are mean±SE, as well as fold difference (Fold Δ) of expression in miR-146a^−/−^ vs. B6. Transcript levels of uninfected cells were very low and similar between strains (not shown). Representative of 3 independent experiments (n≥3 for each experiment).

**Figure 7:**
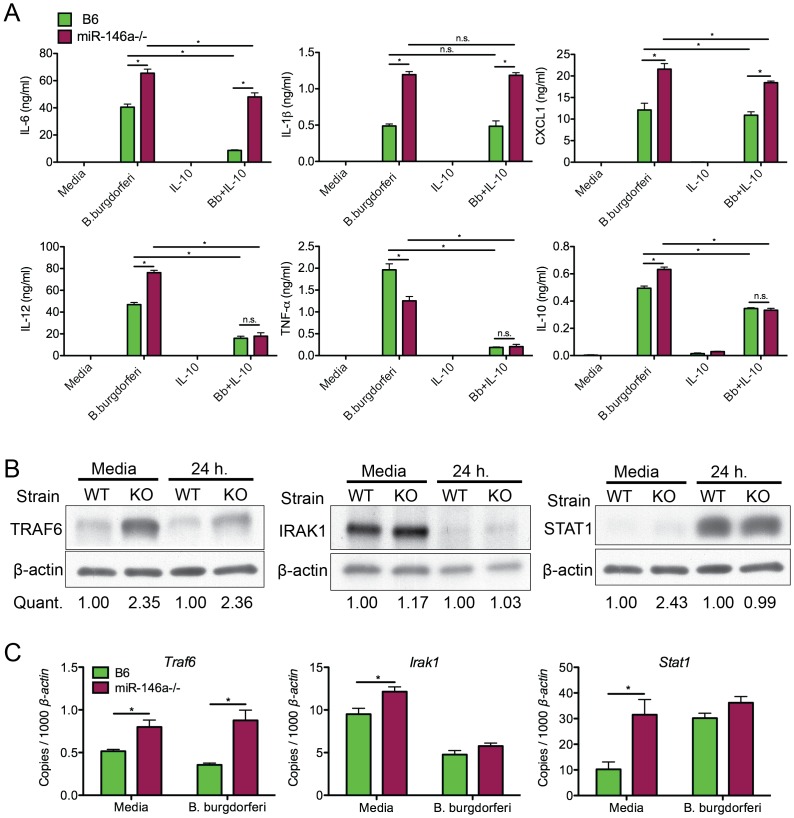
Bone marrow-derived macrophages from B6 miR-146a^−/−^ mice are hyper-responsive to *B. burgdorferi* and have elevated levels of TRAF6. (A) Analysis of cytokine secretion of *B. burgdorferi*-treated BMDMs. Bone marrow-derived macrophages (BMDMs) from B6 (black) or B6 miR-146a^−/−^ (white) mice were pre-treated for 4 hours in the presence or absence of recombinant mouse IL-10. Following pretreatment, cells were stimulated with *B. burgdorferi* for 24 hours. Cell supernatant was collected and secretion of IL-6, IL-1β, CXCL1, IL-12, TNF-α and IL-10 was measured by ELISA. (B) Immunoblot analysis of TRAF6, IRAK1 and STAT1 in *B. burgdorferi*-treated BMDMs from B6 (WT) or B6 miR-146a^−/−^ (KO) mice. BMDMs were stimulated with media alone or *B. burgdorferi* for 24 hours and cells were lysed using NP-40. Quantification was determined based on band intensity, normalized to β-actin, with B6 (WT) value set to 1. (C) *Traf6, Irak1 and Stat1* mRNA levels were quantified using qRT-PCR, normalized to *β-actin*. Significant differences between groups by ANOVA followed by Tukey's post-hoc test are indicated (*p<0.05). Representative of at least 2 independent experiments (n = 3–4 for each group).

We also measured levels of several NF-κB-inducible cytokines by ELISA in cell supernatant from both B6 and B6 miR-146a^−/−^ BMDMs at 24 hours post-stimulation, including TNFα IL-1β, IL-6 and IL-12, CXCL1 and IL-10 (Figure 7A). After 24 hours of treatment with *B. burgdorferi*, three cytokines, IL-1β, IL-6 and IL-12, and the neutrophil chemokine CXCL1, were more abundant in B6 miR-146a^−/−^ cell supernatant than in B6 cell supernatant, consistent with hyperactive NF-κB activation and transcript analysis (Table 3). Interestingly, TNFα, an early-response NF-κB cytokine, did not share this trend, which may be due to the relatively late time point used for this analysis [65]. Production of IL-10 was robust in both strains, although somewhat greater in miR-146a-deficient BMDMs.

Previous work from our laboratory showed that many macrophages are IL-10 producers in joints of B6 mice [48]. Also, macrophages produce high levels of IL-10 when treated with *B. burgdorferi* in vitro, which is important in regulating bacterial persistence [49] and immune response [46], [66]–[68]. Data from Figure 6B also showed that many macrophages in joints of infected B6 and B6 miR-146a^−/−^ mice express the alternatively activated macrophage marker MRC1. While it is difficult to accurately determine the range of macrophage phenotypes present in joints, we used BMDMs pretreated with IL-10 as an *in vitro* model to study miR-146a effects on IL-10-stimulated macrophages. BMDMs were treated with 1ng/ml IL-10 for 4 hours prior to 24-hour *B. burgdorferi* stimulation. Surprisingly, while pretreatment with IL-10 led to an approximately 80% reduction in IL-6 production in B6 BMDMs, IL-10-mediated suppression of IL-6 in B6 miR-146a^−/−^ BMDMs was drastically reduced, with only ∼20% decrease in IL-6 production after IL-10 pretreatment, indicating that IL-10 was unable to effectively suppress IL-6 expression in the absence of miR-146a. These data are consistent with *in vivo* data showing consistently elevated IL-6 protein in serum from 4 week-infected B6 miR-146a^−/−^ mice in Figure 4. However, IL-10 pretreatment did lead to significantly reduced IL-12 and TNFα production in both strains, as well as high production of IL-10, after *B. burgdorferi* treatment, consistent with an anti-inflammatory M2-like phenotype. Both IL-1β and CXCL1 remained higher in B6 miR-146a^−/−^ BMDMs compared to B6 BMDMs, although IL-1β levels were unaffected, and CXCL1 levels were modestly reduced by IL-10 pretreatment. Importantly, levels of IL-12, TNF-α and IL-10 were very similar between the two strains, suggesting there was no miR-146a-mediated defect in M2 polarization in response to IL-10 pretreatment. This is consistent with *in vivo* observations, where TNFα, IL-12 and IFNγ serum protein levels were not significantly elevated in B6 miR-146a^−/−^ mice at 4 weeks post-infection, relative to B6 mice (Figure 4B).

The role of microRNAs, including miR-146a, during inflammatory responses involves suppressing distinct mRNA targets, depending on cell type [69]. It was therefore important to determine the mRNA target most affected at the protein level by the presence or absence of miR-146a in BMDMs. Immunoblot analysis was performed on protein extracts from B6 and B6 miR-146a^−/−^ BMDMs treated for 24 hours with *B. burgdorferi* to measure protein levels of three targets of miR-146a, TRAF6, IRAK1 and STAT1 (Figure 7B). TRAF6 protein expression was elevated over two-fold in both resting and stimulated B6 miR-146a^−/−^ BMDMs compared to B6, while protein levels of IRAK1 were similar between strains. STAT1 protein was also higher in resting B6 miR-146a^−/−^ BMDMs compared to B6, but this difference between strains was not observed after 24 hours stimulation. Transcript analysis of *Traf6*, *Irak1* and *Stat1* also show this trend (Figure 7C). It is interesting that in the case of TRAF6, the difference observed at the protein level was greater than that seen at the transcript level, where transcripts were typically only 30–50% greater in B6 miR-146a^−/−^ BMDMs vs. B6 BMDMs, suggesting that miR-146a effect on translational inhibition is more pronounced than its effect on mRNA stability. This is consistent with a growing body of evidence suggesting that microRNA-mediated translational repression is dependent on inhibition of translation initiation, rather than mRNA degradation [70], [71]. The difference between protein and transcript levels of these three genes (Figure 7B, C) strongly suggests that posttranscriptional regulatory mechanisms including, but not limited to, microRNA-mediated repression, play an important role in determining cellular protein levels. STAT1 is known to be regulated by a large number of posttranslational modifications that affect function [72]. Both STAT1 and IRAK1 protein levels have been shown to be tightly regulated through ubiquitin E3 ligase-directed degradation [73], [74]. In the case of IRAK1 and STAT1, these data suggest that miR-146a-independent regulatory mechanisms seem to be dominant compared to miR-146a-mediated regulation. Taken together, TRAF6 protein levels appear to be the most sensitive to the presence or absence of miR-146a in myeloid cells, and imply miR-146a-mediated translational repression of TRAF6 is required to properly regulate production of NF-κB-induced cytokines in response to *B. burgdorferi*. The lack of difference in STAT1 protein level is also consistent with a failure to observe significant differences between B6 and B6 miR-146a^−/−^ mice in the IFN response (Figures 4&5).

### Macrophages lacking miR-146a have increased phagocytic activity

**Figure 8:**
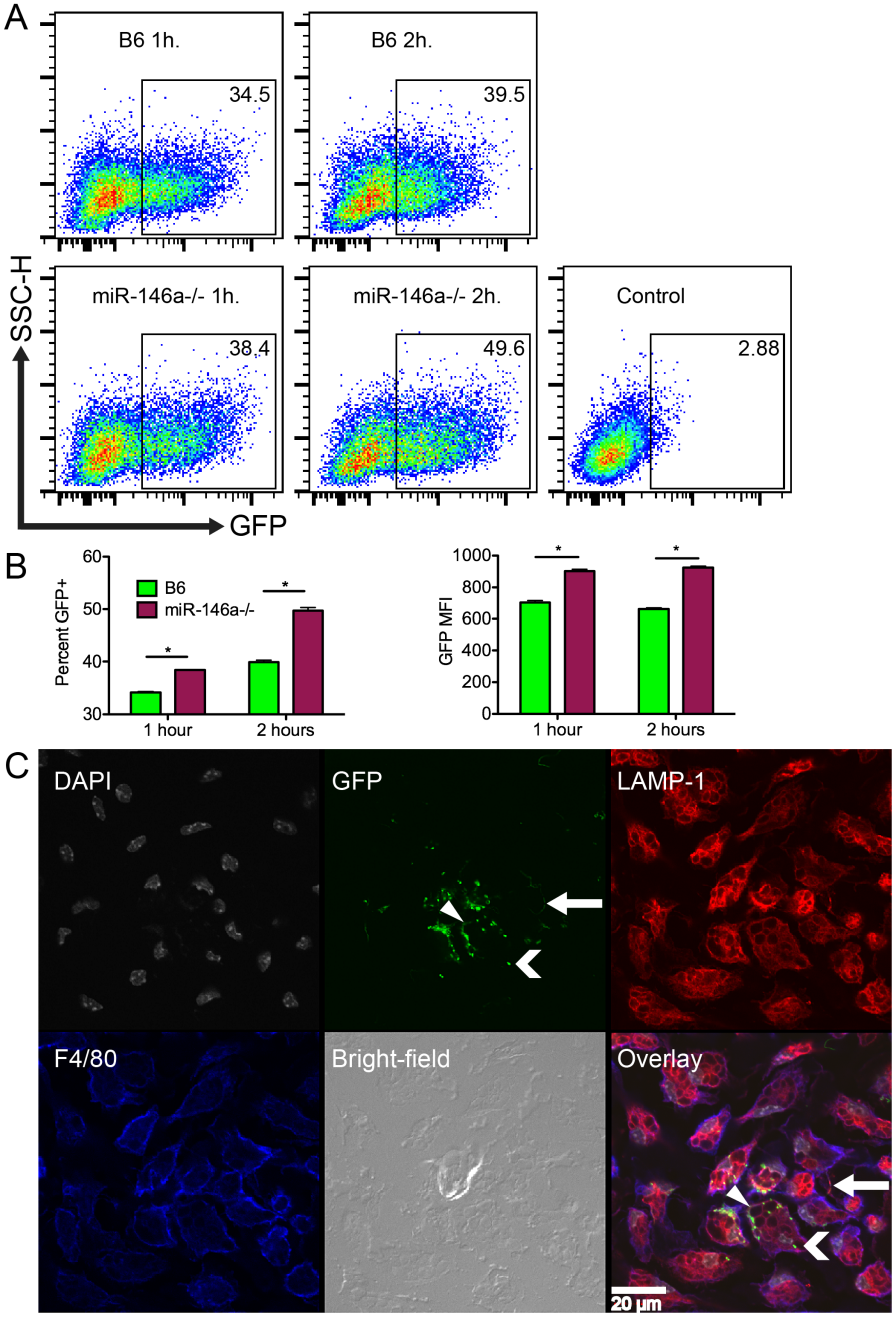
B6 miR-146a^−/−^ peritoneal macrophages exhibit increased phagocytic activity. (A) Representative plots of peritoneal macrophages isolated from B6 or B6 miR-146a^−/−^ mice and incubated with GFP-*B. burgdorferi* for 1 or 2 hours at 10∶1 MOI. Box indicates GFP+ fraction, and number in top-right corner is percent GFP+. Control is using cells only. (B) Mean percent GFP+ and GFP mean fluorescence intensity for flow analysis shown in (A). Representative of two independent experiments (n = 3 for each experiment). Significant differences between groups by ANOVA followed by Tukey's post-hoc test are indicated (*p<0.05). (C) Confocal images of B6 peritoneal macrophages incubated with GFP-*B. burgdorferi* for 1 hour at 100∶1 MOI. Panels are (from top-left) cell nuclei (gray, DAPI), GFP-*B. burgdorferi* (green), lysosomes (red, LAMP-1), cell membrane (blue, F4/80), bright-field and overlaid images. White bar indicates scale. White arrow shows a bacterium associated with a macrophage pseudopod, chevron indicates bright GFP puncta associated with intracellular lysosomes, and triangle indicates a cell surface-associated bacterium. Representative of two biological replicates. Similar images of B6 miR-146a^−/−^ macrophages can be found in Figure S2.

One possible explanation for reduced numbers of *B. burgdorferi* in joints of infected B6 miR-146a^−/−^ mice is that macrophages lacking miR-146a are more highly phagocytic. In order to measure phagocytic activity, peritoneal macrophages were collected from B6 and B6 mir-146a^−/−^ mice and stimulated with GFP-labeled *B. burgdorferi* for 1 or 2 hours at 10∶1 multiplicity of infection (MOI). Phagocytosis of GFP-*B. burgdorferi* was measured by flow cytometry (Figure 8A&B). At both 1 and 2 hours post-stimulation, peritoneal macrophages lacking miR-146a had significantly higher numbers of GFP+ cells, as well as a higher mean fluorescence intensity (MFI) for GFP in GFP+ macrophages. These data suggest that there are more B6 miR-146a^−/−^ peritoneal macrophages associated with higher numbers of bacteria than wild-type cells.

Flow cytometry was unable to distinguish localization of the cell-associated bacteria. To determine whether GFP-*B. burgdorferi* were intracellular or adhering to the cell surface, confocal microscopy was used to visualize the bacteria associated with peritoneal macrophages. Peritoneal macrophages were stimulated with GFP-*B. burgdorferi* at 100∶1 MOI for 1 hour and stained for the lysosomal protein LAMP1 (red), the macrophage-specific surface protein F4/80 (blue), and nuclei were stained with DAPI (gray, Figure 8C). Bacteria were visible adhering to cell surface (white triangle), inside macrophage pseudopodia (white arrow) and inside cells associated with LAMP1 (white chevron). While bacteria adhering to the cell surface and inside pseudopodia had a spirochetal shape, bacteria associated with lysosomes were amorphous, and formed bright GFP puncta, indicative of bacterial degradation. These bright GFP puncta were predominant throughout the entire sample, as represented in the image in Figure 8C for B6 mice, and in Figure S2 for B6 miR-146a^−/−^ mice. This indicates that phagocytosis occurs very rapidly as previously reported [65], and the flow cytometry analysis infers that miR-146a modulates the level of phagocytic activity. Although the mechanism is unknown, similar transcript levels were seen for TLR2, CD14, as well as the scavenger receptor MARCO (data not shown), which have been recently implicated in *B. burgdorferi* uptake [75]–[78]. Previous reports showing phagocytosis influencing cytokine production in human mononuclear cells [78], and B6 MyD88^−/−^ BMDMs being defective in bacterial internalization [79], are consistent with B6 miR-146a^−/−^ BMDMs having elevated cytokine production and enhanced phagocytic activity (Figure 7, Table 3, Figure 8). While more research is necessary to elucidate this mechanism, these data suggest that B6 miR-146a^−/−^ macrophages have enhanced phagocytosis, and may help explain why joint tissue from B6 miR-146a^−/−^ mice contains fewer numbers of spirochetes (Figure 2C).

## Discussion

**Figure 9:**
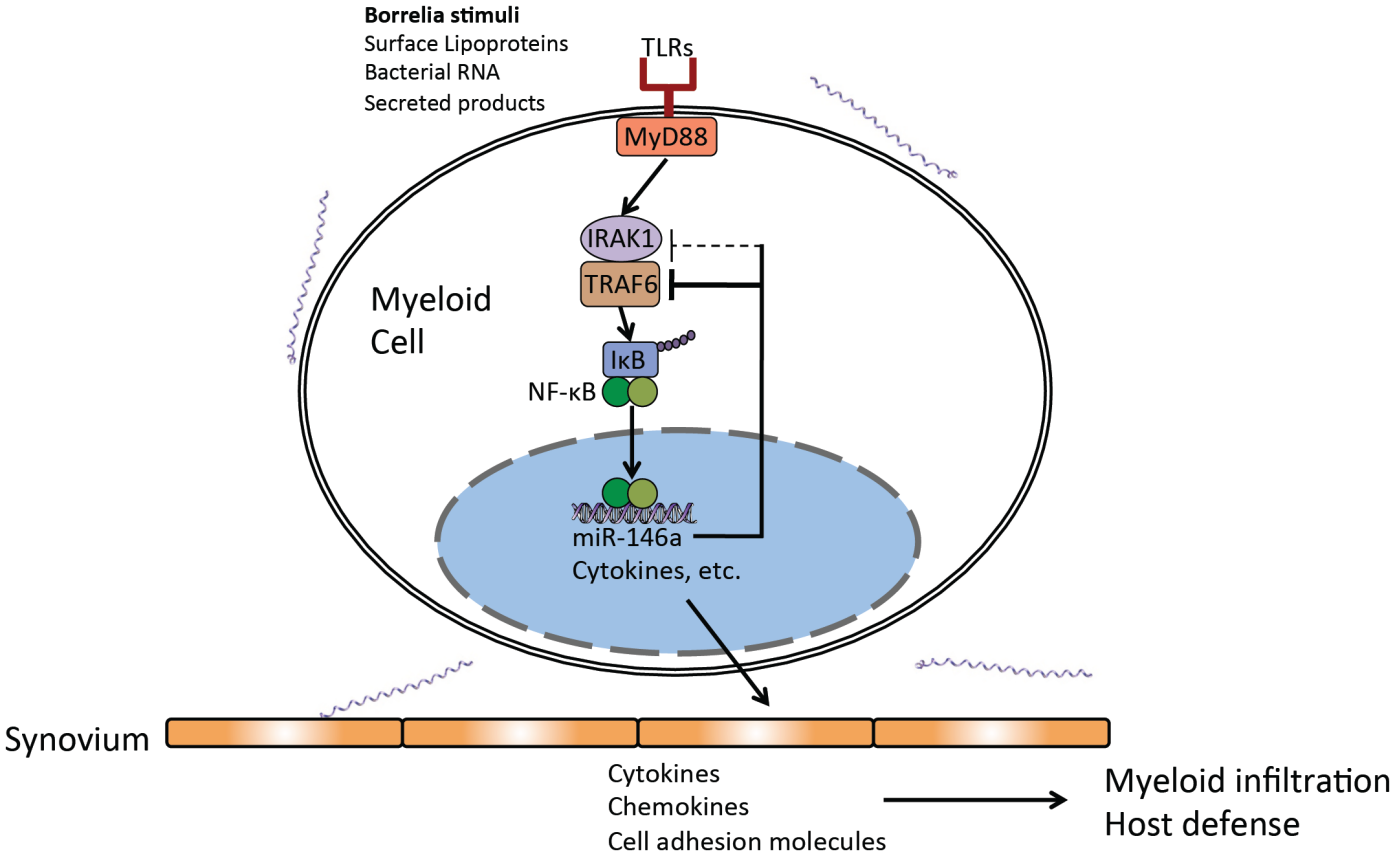
Model of miR-146a function as a suppressor of arthritis during persistent *B. burgdorferi* infection. Regulatory role of miR-146a in myeloid cells present in joint tissue as a negative regulator of Toll-like receptor (TLR) signaling through suppression of adaptor proteins, particularly TRAF6.

These data have allowed us to generate a model (Figure 9) where miR-146a is upregulated during *B. burgdorferi* infection, and acts as a nonredundant suppressor of inflammation and arthritis (Figures 1–2). Interestingly, lack of miR-146a had no effect on heart inflammation and carditis (Figure 3), indicating fundamental differences between arthritis and carditis development. Differences in carditis severity between B6 and C3H mice are believed to be closely associated with differences in bacterial dissemination and clearance between the two strains [80]. This is consistent with the positive correlation between bacterial numbers and heart lesion severity in B6, B6 miR-146a^−/−^ and C3H mice (Figure 3A–B), and with previous reports showing no correlation between quantitative trait loci associated with arthritis severity and bacterial numbers in heart tissue [81], [82]. Importantly, differential contribution of NF-κB regulation was not predicted from studies with mice deficient in TLR2 and MyD88, as both hearts and joint tissues displayed increased presence of *B. burgdorferi* [18], [19], [83].

Numerous studies have revealed different mechanisms of pathogenesis in carditis development and differing contributions of innate and adaptive responses in bacterial clearance and resolution of carditis and arthritis. For example, although antibody response plays an essential role in resolution of arthritis, greater roles of CD4+ T cells and iNKT cells as sources of IFNγ are reported in protection and resolution of Lyme carditis [55], [60], [84]–[86]. Other gene knockout and cytokine blocking studies have shown tissue-specific effects of IL-10 [46] and chemokines [51], [62] on arthritis and carditis severity. These results suggest future microRNA studies on carditis should focus on those miRs known to influence the balance of CD4+ T cells [87] and iNKT cell function, such as miR-150 and miR-181a/b [88]–[90].

Myeloid cells respond to a variety of *Borrelia* stimuli through TLRs that lead to activation of NF-κB and upregulation of hundreds of genes involved in controlling infection and initiating the adaptive response. miR-146a is also upregulated, and is an important check on the amplitude and duration of the NF-κB response. In the absence of this microRNA, this response is dysregulated, leading to increased transcription of certain NF-κB-inducible cytokines and chemokines in infected joint tissue, primarily late in infection (Figures 4–5). Myeloid cells exhibit excessive proliferation and infiltration into joint tissue of B6 miR-146a^−/−^ mice, have increased phagocytic activity and produce excess cytokines such as IL-1β, IL-6 and CXCL1, leading to inflammation of synovial tissue and arthritis development (Figures 6–8). Regulation of the inflammatory response via a miR-146a-mediated negative feedback loop is critical for resolution of the NF-κB response during the persistent phase of infection, and mice lacking this miRNA are poised to develop arthritis upon infection with *B. burgdorferi*.

NF-κB activation in response to *B. burgdorferi* infection is a double-edged sword. On one hand, NF-κB activation is critical in mounting an effective immune response to control infection; on the other hand, dysregulated activation leads to inflammation and arthritis. Because of the dual nature of NF-κB in inflammation and host defense, decoupling these two roles has been difficult. Knockout models using B6 TLR2^−/−^ or MyD88^−/−^ mice have shown the important role of NF-κB in host defense, but because these mice have such a severe innate defect in bacterial defense, elucidating the role of NF-κB in arthritis using these models has remained difficult. The B6 miR-146a^−/−^ mouse model of Lyme arthritis is unique in that it effectively decouples these two roles, leaving the bactericidal function intact while increasing the amplitude of proinflammatory NF-κB activation. This has allowed us to identify its role in arthritis development, independent of its role in host defense, and suggests that miR-146a could be a valuable therapeutic target for control of inflammation without compromising ability to clear an infection.

MicroRNAs are a unique class of regulatory molecules. Unlike transcription factors, they do not act as on/off switches, rather they function as “fine tuners” of gene expression [26]. We have taken advantage of this property to decouple the roles of NF-κB in host defense and inflammation. Young B6 miR-146a^−/−^ mice are phenotypically similar to wild-type B6 mice, and it is only upon chronic exposure to inflammatory stimuli that immunological defects are seen [29]. Consistent with this, endotoxin tolerance is highly dependent upon miR-146a expression in THP-1 cells [91]. Using Lyme arthritis as a model, we have shown that mice lacking this key miRNA fail to adequately maintain immune homeostasis, and develop inflammatory arthritis during a chronic bacterial infection (Figure 2, Table 2).

This model is also distinct from other mouse models of Lyme arthritis. For example, C3H mice exhibit a robust Type I IFN expression profile early in infection, which contributes to arthritis, and is absent in the mildly arthritic B6 mouse [43], [44], [47]. This IFN response was absent miR-146a^−/−^ mice, similar to B6. Furthermore, B6 IL10^−/−^ mice, a model for Th1-mediated arthritis, have a very pronounced IFNγ signature beginning at 14 days post-infection that persists for several weeks [48]. This pattern was also not observed in the arthritic B6 miR-146a^−/−^ mice (Figures 4–5, Figure S1). Additionally, while B6 miR-146a^−/−^ and B6 IL10^−/−^ mice both exhibit increased bacterial clearance, likely due to an enhanced myeloid response to phagocytosis of bacteria [63], only B6 IL10^−/−^ mice show enhanced antibody response [49]. It was somewhat surprising that B6 miR-146a^−/−^ mice did not exhibit a strong T-cell-mediated phenotype, based on parameters tested, since other studies have shown an important role of miR-146a in regulating Th1 responses [31], [92]. It is possible that the elevated myeloid response could eventually lead to a dysregulated T-cell response in some cases. Indeed, several B6 miR-146a^−/−^ mice did have elevated serum IFNγ at 4 weeks post-infection, although this was the exception rather than the rule, and average levels did not achieve statistical significance compared to wild-type mice (Figure 4C). It may also be possible that robust production of IL-10 seen in B6 miR-146a^−/−^ mice is sufficient to suppress any T-cell dysregulation due to lack of miR-146a. Nevertheless, the results of this study show that arthritis is influenced principally by hyperactive myeloid cell activation.

The role of miR-146a in regulating NF-κB activation was consistent with the observed defect in downregulation of NF-κB-dependent cytokines and chemokines *IL-1β*, *IL-6*, *Cxcl1* and *Cxcl2*, in B6 miR-146a^−/−^ mice at 4 weeks post-infection (Figure 4). Dysregulation of *Cxcl1* in these mice was particularly interesting because previous studies have shown that C3H mice lacking CXCL1 have reduced neutrophil infiltration and arthritis [61], [62]. This neutrophil chemokine is tightly regulated both at the transcriptional and posttranscriptional level by both TLR dependent and cytokine dependent mechanisms [93]. Data from Figure 7 suggest that excess cytokine production by B6 miR-146a^−/−^ macrophages may lead to enhanced CXCL1 production by resident cells *in vivo*. Therefore, miR-146a, expressed primarily in leukocytes [29], likely has cell-extrinsic effects on nonhematopoietic cell function and arthritis development. Recently, IL-6 has been shown to be an important downstream target of miR-146a in regulating hematopoiesis and myeloproliferation [29]. This is consistent with increased IL-6 production shown in Figure 4 and Figure 7, and corresponding increase in myeloid cell infiltration into joint tissue (Figure 6). Thus, miR-146a-mediated regulation of several cytokines and chemokines likely has a combined effect on inflammatory responses.

Increased phagocytic activity, as well as elevated IL-1β production (Figures 7–8) point to a previously unrecognized role of miR-146a in phagocytosis and caspase-1 activation. While this role remains to be elucidated, previous research has shown that *B. burgdorferi* induces caspase-1-dependent IL-1β production, and caspase-1 is important for inflammatory cell influx into joint tissue [94]. Additionally, phagocytosis of live *B. burgdorferi* is a potent activator of IL-1 β in human PBMCs [95].

Targets of miR-146a have been studied in many cell types, and it is becoming increasingly evident that the modulatory effect of miR-146a is dependent on cell type and physiological condition. For example, STAT1 appears to be an important miR-146a target in regulatory T-cells [92], and IRAK1 and TRAF6 both appear to be important miR-146a targets in splenocytes [31] and human monocytes [30]. This study highlights the particular role of miR-146a targeting TRAF6 in myeloid cells (Figure 7), indicating that miR-146a function is, to a certain degree, cell type-specific. Importantly, several observations in the B6 miR-146a^−/−^ mouse model are recapitulated in Lyme disease patients. Joint fluid and synovial tissue from antibiotic-refractory Lyme arthritis patients contain higher levels of IL-6 and IL-1β, as well as Th1 cytokines and chemokines, compared with patients whose arthritis is resolved after antibiotic treatment, and IL-1β remains elevated in these treatment-refractory patients long after antibiotic therapy [96], [97]. Thus, the B6 miR-146a^−/−^ model of Lyme arthritis could be a useful tool in further understanding how regulation of NF-κB is related to Lyme disease pathogenesis.

## Materials and Methods

### Ethics statement

Mice were housed in the University of Utah Comparative Medicine Center (Salt Lake City, UT), following strict adherence to the guidelines according to the National Institutes of Health for the care and use of laboratory animals, as described in the Guide for the Care and Use of Laboratory Animals, 8^th^ Edition. Protocols conducted in this study were approved and carried out in accordance to the University of Utah Institutional Animal Care and Use Committee (Protocol Number 12-01005). Mouse experiments were performed under isofluorane anesthesia, and every effort was made to minimize suffering.

### Mice, bacterial cultures and infections, and assessment of arthritis severity

C3H, C57BL/6 and B6.129P2-*IL-10tm1Cgn*/J (B6 IL10^−/−^) mice were obtained from Jackson Laboratories. B6 miR-146a^−/−^ KO mice on a pure C57BL/6 background were generated as described [28]. Mice were infected with 2×10^4^ *B. burgdorferi* strain N40 (provided by S. Barthold, University of California, Davis, CA) by intradermal injection into the skin of the back. Infection was confirmed in mice sacrificed before 14 d of infection by culturing bladder tissue in BSK II media containing 6% rabbit serum (Sigma-Aldrich), phosphamycin and rifampicin. ELISA quantification of *B. burgdorferi*-specific IgM and IgG concentrations was used to confirm infection in mice sacrificed at and after 14 d of infection as described [17]. Ankle measurements were obtained using a metric caliper. Rear ankle joints were prepared for assessment of histopathology by removal of the skin and fixation of tissue in 10% neutral buffered formalin. Decalcified joints were embedded in paraffin, sectioned at 3 µm, and stained with H&E. Each slide was scored from 0 to 5 for various aspects of disease, including polymorphonuclear leukocyte (PMN) and mononuclear cell (lymphocytes, monocytes, macrophages) infiltration into inflammatory processes, tendon sheath thickening (hypertrophy and hyperplasia of surface cells and/or underlying dense sheets of cells resembling immature fibroblasts, synoviocytes, and/or granulation tissue), reactive/reparative responses (periosteal hyperplasia and new bone formation and remodeling), and overall lesion (composite score based on all lesions observed in 6–8 sections per joint), with 5 representing the most severe lesion, and 0 representing no lesion. Ankle measurements and arthritic lesions were assessed in coded samples.

Hearts of B6, B6 miR-146a^−/−^ and C3H mice were assessed for carditis by histopathologic evaluation at 3 weeks post-infection. Hearts were fixed in 10% neutral buffered formalin, embedded in paraffin and sectioned at 3 µm, and stained with H&E. Lesion scoring was performed in a blinded fashion based on a composite of 11 sections per sample, with a score of 5 representing the maximum lesion and 0 representing no lesion.

### miRNA microarray

Microarray analysis was performed with the assistance of the University of Utah Microarray and Bioinformatics core facilities. Whole joint RNA was purified from mouse joints (3–4 mice per sample group) using miRNeasy kit (Qiagen). RNA quality was determined using a Bioanalyzer 2100 and RNA 6000 Nano Chip (Agilent Technologies). Agilent Mouse miRNA microarray v2 (8×15k) was hybridized with Cyanine-3 labeled miRNA (using 100 ng total RNA) using the Agilent one-color GE hybridization and wash kit. Slides were scanned in a G2505C Microarray Scanner at 2 um resolution (Agilent Technologies). TIF files generated from the scanned microarray image were analyzed in the Agilent Feature Extraction Software (v.10.5), which was used to calculate feature intensities, background measurements and statistical analyses. Data sets for each biological sample were then filtered and log(2) transformed using an in-house java script, and were uploaded into Geospiza GeneSifter Analysis Edition (Perkin Elmer). Pair-wise analysis between groups was performed using a quality cutoff for both groups of 1, normalizing to median values, with a cutoff value of 2-fold change compared to uninfected controls.

### Isolation of DNA from ear tissue and quantification of *B. burgdorferi*

DNA was prepared from ear tissues frozen at the time of sacrifice. Tissue was incubated in 50 mM NaOH for 1 hour at 93°C and neutralized with 1M Tris (pH 8). Quantification of *B. burgdorferi recA* normalized to the mouse *nidogen* was performed using a Roche LC-480 using previously published primers [18].

### Preparation of single-cell suspensions from mouse tissue

Single-cell suspensions were prepared as previously described [43]. Skin was removed from rear ankle joints and digested for 1 h at 37°C in RPMI 1640 containing 0.2 mg/ml purified enzyme blend for tissue dissociation (Roche) and 100 µg/ml DNase I (Sigma-Aldrich), following partial removal of tissue from bone using 20-gauge syringe needles. Single cell suspension was filtered through a 100 µm cell strainer and red blood cells were lysed using ammonium-chloride-potassium (ACK) lysing buffer.

### Isolation of RNA and quantitative RT-PCR

For all experiments examining expression in heart and joint tissue, RNA was purified from the heart or tibiotarsal joints with the skin removed. Tissue was immediately immersed in RNA stabilization solution (Qiagen) and stored at −80°C. Total RNA was recovered from homogenized tissue using the miRNeasy kit (Qiagen). For FACS-sorted cell populations, sorted cells were collected directly in flow tubes containing 0.5 ml RNA stabilization solution (Qiagen) and RNA was recovered using the miRNeasy kit (Qiagen). RNA from BMDMs was recovered using guanidium thiocyanate-phenol-chloroform extraction reagent (Invitrogen). RNA recovered from tissue and cells was reverse transcribed, and transcripts were quantified using a Roche LC-480 according to our previously described protocols [47]. For mature miRNA quantification, cDNA was synthesized using the mercury Locked Nucleic Acid Universal RT microRNA PCR, Polyadenylation and cDNA synthesis kit (Exiqon), and miR-146a, 5S rRNA Locked Nucleic Acid primer sets were used (Exiqon) to quantify miRNA using a Roche LC-480. Other primer sequences used in this study were as follows: *Itgam* (CD11b) FWD (5′-CCTTCATCAACACAACCAGAGTGG-3′) REV (5′- CGAGGTGCTCCTAAAACCAAGC-3′), *Irak1* FWD (5′-TGTGCCGCTTCTACAAAGTG-3′) REV (5′-TGTGAACGAGGTCAGCTACG-3′), *Traf6* FWD (5′-AAGCCTGCATCATCAAATCC-3′) REV (5′-CTGGCACTTCTGGAAAGGAC-3′). Primer sequences for *B. burgdorferi 16S rRNA*, *β-actin*, *Il1β*, *Stat1*, *Tnfa*, *Oasl2* [47], *Vα14*, *F4/80* [48] *Il10*, *Ifng*, *Cxcl10*, *Il6* [44] *Cxcl1*, *Cxcl2*, *Pecam1* (CD29), and *Ptprc* (CD45) [43] can be found in indicated citations.

### Flow cytometry

All flow cytometry data were analyzed using FlowJo (v.5) software. Sorting experiments were performed using a BD FACSAria II. All other FACS data were collected on a BD LSRII flow cytometer. 7-aminoactinomycin D (eBioscience) or DAPI (Invitrogen) was used in all experiments, and dead cells and cell doublets were excluded from analyses. All Abs used for flow cytometry were purchased from either BioLegend or eBioscience. Unconjugated F_c_ blocking Ab (clone 93; BioLegend) was included in all Ab-labeling experiments. Position of gates for sorting and analysis was based on analysis of appropriate isotype controls. Fluorochrome-conjugated Abs and isotype controls used in this study were as follows: APC/Cy7-conjugated anti-CD11b (M1/70) and anti-CD45.2 (104); FITC-conjugated anti-CD8a (53-6.7), anti-CD11b (M1/70) and anti-Gr-1 (RB6-8C5); PerCP/Cy5.5-conjugated anti-Ly6C (HK1.4), anti-CD4 (RM4-4) and anti-CD31 (390); PE-conjugated anti-F4/80 (BM8) anti-LAMP-1 (1D4B) and anti-NK1.1 (PK136); PE/Cy-7–conjugated anti-CD4 (GK1.5) and anti-TCR β (H57-597); APC-conjugated anti-CD206 (MMR) and anti-F4/80 (BM8); and Brilliant Violet 605-conjugated anti-B220 (RA3-6B2). Confirmation of cell sorting efficiency was performed using qRT-PCR of surface markers used.

### Bone marrow-derived macrophage stimulation

Bone marrow-derived macrophages (BMDMs) were isolated from the femurs and tibias of mice, as previously described [98]. Macrophage cultures were plated in 12-well plate at a density of 6×10^5^/ml in media containing the serum replacement Nutridoma (Roche) and stimulated with live *B. burgdorferi* cN40 (7.5×10^6^/ml). Priming of macrophages was performed by pre-incubating cells with 1 ng/ml mouse recombinant IL-10 for 4 hours prior to addition of *B. burgdorferi*. After 24 hours, cell supernatants were collected and analyzed by enzyme-linked immunosorbent assay (ELISA). For expression analysis, RNA was collected from cells at 6 hours and 24 hours post-stimulation, and mRNA quantification was performed by qRT-PCR using methods described above.

### ELISA analysis of mouse serum and cell supernatant

Blood from mice was obtained by submandibular puncture at the time of euthanasia. Blood was allowed to clot, centrifuged, and serum was collected and stored at −20°C prior to analysis. Cell supernatant was used immediately or stored at −20°C prior to analysis. Cytokine concentration in serum samples and cell supernatant was detected by sandwich ELISA using capture and biotinylated antibodies against mouse IL-1β (clones B122 and Poly5158, Biolegend), IL-6 (clones MP5-20F3 and MP5-32C11, BD Biosciences), IL-10 (clones JESS-2A5 and SXC-1, BD Biosciences) IL-12 (clones C15.6 and C17.8, BD Biosciences).IFNγ (clones R46A2 and XMG1.2, BD Biosciences), TNFα (clones G281-2626 and MP6-XT3, BD Biosciences) and CXCL1 (clone 48415 and Cat BAF453, R&D Systems).

### Immunoblot analysis

Cells were washed and lysed at 4°C. with NP-40 lysis buffer (0.5% NP-40) for 1 hour followed by boiling for 5 minutes in SDS sample buffer. Protein concentration was measured using a BCA protein assay (Thermo Scientific). Proteins were separated by polyacrylamide gel electrophoresis (PAGE) and transferred overnight at 4°C. onto an Immobilon-P membrane (Millipore). Membrane was blocked with 5% milk in TBST and stained with the following antibodies: rabbit anti-TRAF6 (clone H-274, Santa Cruz), rabbit anti-IRAK1 (clone D51G7, Cell Signaling) rabbit anti-STAT1 (Cell Signaling #H9172S) and rabbit anti-β-actin (clone 13E5, Cell Signaling) as a loading control. Horseradish peroxidase-conjugated goat anti-rabbit IgG (BioRad) was used as a secondary antibody prior to incubation with enhanced chemoluminescent substrate (Thermo Scientific). Membrane was exposed to autoradiography film (GeneMate) and developed using a medical film processor (SRX-701, Konica Minolta).

### Phagocytosis assay

Mice received an intraperitoneal (IP) injection of 3 ml of 3% thioglycollate 4 days prior to harvesting of peritoneal macrophages. Macrophages were removed from sacrificed mice by IP injection of 5 ml ice-cold PBS. Red blood cells were lysed using ACK lysis buffer. 5×10^5^ cells were allowed to adhere to a 12-well plate in RPMI+10% FBS for 4 hours, after which cells were washed and unadhered cells removed. 5×10^6^ *B. burgdorferi* strain N40 constitutively expressing GFP under the flaB promoter [99] (a gift from Dr. Jay Carroll) were added to cells in RPMI.B (75% RPMI+10% FBS+25% BSKII), as described [100], plates were centrifuged at 500 g for 5 minutes and incubated for 1 or 2 hours. After which cells were washed gently 3× in warm PBS and gently removed from the plate using a cell scraper. Cells were washed 2× with ice-cold PBS and supernatant discarded following centrifugation. Washed cells were then resuspended in flow buffer and analyzed by flow cytometry using a BD LSRII flow cytometer. As a negative control, untreated cells and cells incubated with unlabeled *B. burgdorferi* N40 were used.

### Confocal microscopy

Peritoneal macrophages were harvested as described above and allowed to adhere to the surface of etched microscope cover slides for 4 hours. Cells were incubated with GFP-*B. burgdorferi* for 1 hour at a 100∶1 MOI as described above, followed by 4× washes with warm PBS, fixed in 4% paraformaldehyde, and incubated with antibody blocking solution (3% BSA 0.05% milk 0.2% Tween20 in PBS) for 1 hour at RT. Cells were then stained with PE-conjugated LAMP-1, APC-conjugated F4/80 and DAPI for 1 hour in antibody solution (1% BSA 0.02% Tween20 in PBS), washed and mounted onto a glass slide using fluorescent mounting reagent (Calbiochem EMD Millipore). Confocal imaging was performed on a FV1000 inverted confocal microscope (Olympus) using FV10-ASW software (Olympus). Images were taken using a 60× oil lens with a 1024×1024 2× zoom, and captured at a plane dissecting the middle of cell nuclei. All imaging was performed at the University of Utah Cell Imaging Core Facility, with the assistance of Dr. Christopher Rodesh.

### Data and statistical analysis

Microarray data statistical analysis was performed using the Agilent Feature Extraction Software (v.10.5) and Geospiza GeneSifter Analysis Edition (Perkin Elmer), as described. Raw and adjusted p values were derived by Welch's *t* test with Benjamini and Hochberg correction, using a raw p value cutoff of p<0.05 signifying statistical significance. All other graphical data represent the mean ± SEM. Statistical analysis was performed using Prism 5.0c software. Multiple-sample data sets were analyzed by one-way ANOVA with Dunnet's or Tukey's post hoc test for pair-wise comparisons, as appropriate and indicated in figure legends. Two-sample data sets were analyzed by Student *t* test. Categorical data for histopathology was assessed by Mann-Whitney *U* test. Statistical significance indicated in figure legends.

## Supporting Information

Figure S1 **Lymphocyte infiltration into joints of** ***B. burgdorferi*** **-infected mice are similar between WT and miR-146a^−/−^ mice.** Flow cytometry analysis of lymphoid cells released from joint tissue of B6 or B6 miR-146a^−/−^ mice infected with *B. burgdorferi* for 2 or 4 weeks, following gating to exclude debris, dead cells and cell doublets. Cell lineages defined as follows: T cells (CD45+ TCRβ+), B cells (CD45+ B220+), and NK cells (CD45+ NK1.1+). No significance was observed between any groups by ANOVA followed by Tukey's post-hoc test (*p<0.05).
(TIFF)

Figure S2 **Confocal images of B6 miR-146a^−/−^ peritoneal macrophages incubated with GFP-** ***B. burgdorferi*** **.** Panels are (from top-left) cell nuclei (gray, DAPI), GFP-*B. burgdorferi* (green), lysosomes (red, LAMP-1), cell membrane (blue, F4/80), bright-field and overlay fields. White bar indicates scale.
(TIF)

Table S1 **MicroRNAs with greater than 2-fold change in expression in joints of mouse strains, based on Agilent miRNA microarray analysis.** All microRNAs with greater than 2-fold change in expression in *B. burgdorferi*-infected joints of B6, C3H and B6 IL-10^−/−^ mice at one and two weeks post-infection, compared to uninfected controls, based on Agilent mouse miRNA microarray. Shown is gene name, fold-change, direction of fold-change, p-value and adjusted p-value. Significance was determined using Welch's t-test with Benjamini and Hochberg correction (p<0.05, n = 3–4 mice per group).
(XLSX)
