# Supplementary figures and images for: MicroRNA-146a Provides Feedback Regulation of Lyme Arthritis but Not Carditis during Infection with Borrelia burgdorferi
Source: PLoS Pathog. 2014 Jun 26;10(6):e1004212. doi: 10.1371/journal.ppat.1004212 (PMC4072785; doi:10.1371/journal.ppat.1004212)

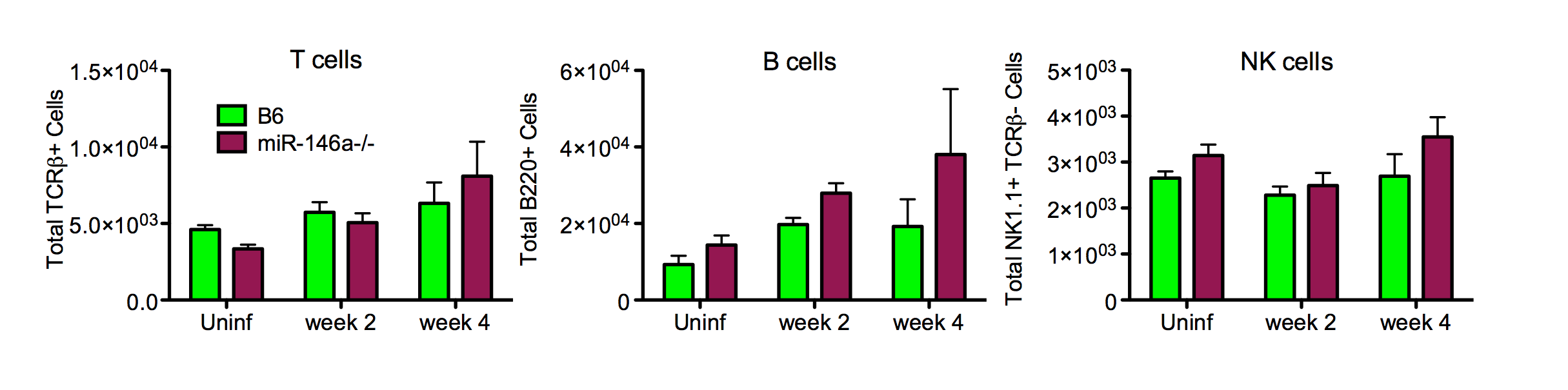

Supplement: Figure S1 — Lymphocyte infiltration into joints of B. burgdorferi -infected mice are similar between WT and miR-146a−/− mice. Flow cytometry analysis of lymphoid cells released from joint tissue of B6 or B6 miR-146a−/− mice infected with B. burgdorferi for 2 or 4 weeks, following gating to exclude debris, dead cells and cell doublets. Cell lineages defined as follows: T cells (CD45+ TCRβ+), B cells (CD45+ B220+), and NK cells (CD45+ NK1.1+). No significance was observed between any groups by ANOVA followed by Tukey's post-hoc test (*p<0.05). (TIFF) [file ppat.1004212.s001.tiff]

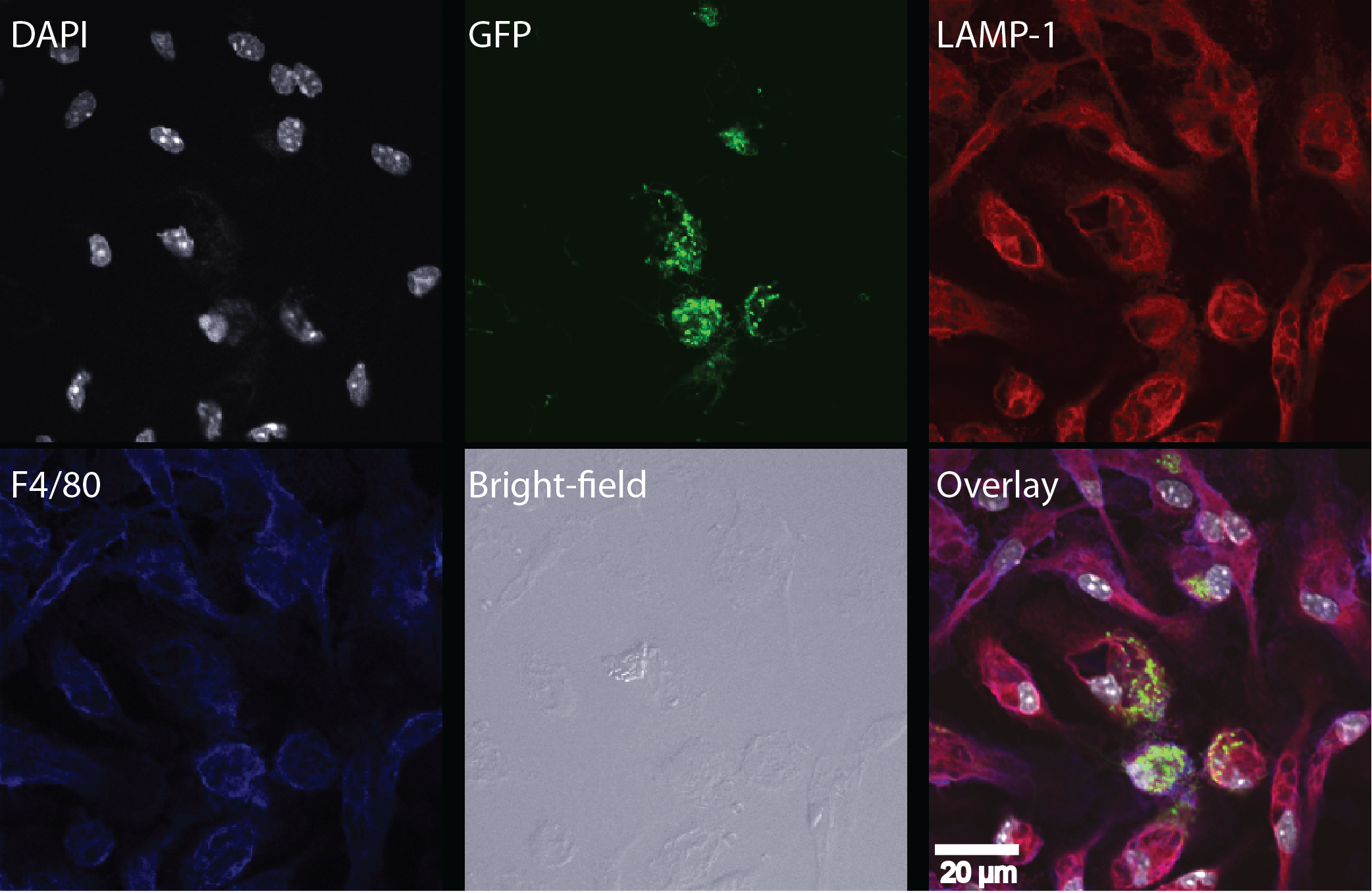

Supplement: Figure S2 — Confocal images of B6 miR-146a−/− peritoneal macrophages incubated with GFP- B. burgdorferi . Panels are (from top-left) cell nuclei (gray, DAPI), GFP-B. burgdorferi (green), lysosomes (red, LAMP-1), cell membrane (blue, F4/80), bright-field and overlay fields. White bar indicates scale. (TIF) [file ppat.1004212.s002.tif]
